# Feasibility of a Complex Setting for Assessing Sleep and Circadian Rhythmicity in a Fragile X Cohort

**DOI:** 10.3389/fpsyt.2020.00361

**Published:** 2020-05-14

**Authors:** Alexander Dueck, Olaf Reis, Manuela Bastian, Lucas van Treeck, Steffen Weirich, Frank Haessler, Andreas Fiedler, Michael Koelch, Christoph Berger

**Affiliations:** ^1^Department for Psychiatry, Neurology, Psychosomatics and Psychotherapy in Childhood and Adolescence, Rostock University Medical Center, Rostock, Germany; ^2^Institute for Clinical Chemistry and Laboratory Medicine, Rostock University Medical Center, Rostock, Germany; ^3^Department of Child and Adolescent Psychiatry, GGP Group, Rostock, Germany; ^4^Department for Pediatric and Adolescent Medicine, Klinikum St. Marien, Amberg, Germany

**Keywords:** Fragile X, sleep, circadian rhythm, melatonin, polysomnography

## Abstract

**Introduction:**

Sleep, circadian rhythms, (mental) health, and development are assumed to be intertwined. However, differentiated and reliable parameters of sleep and circadian rhythms are particularly difficult to assess for Fragile X (FXS) individuals. As those parameters need to be observed in complex settings, the feasibility of measurements for people with FXS was to be proven. Findings from this pilot study can inform further research and help to estimate sample sizes for future studies on FXS patients.

**Methods and Sample:**

Nine individuals (male and female) with full mutation of the FMR1 gene were integrated in the study and underwent a complex measurement including actigraphy, sleep log, and 24-h saliva sampling in order to examine profiles of melatonin and cortisol, and a polysomnography.

**Results:**

Seven actigraphy profiles, eight sleep logs, eight saliva profiles and seven polysomnographic data sets were collected. Complete data were analyzed for six individuals [mean age 14.87 years (SD 4.12), mean BMI 25.90 (SD 4.44)] were collected. No drop outs due to the constraints of the assessment were registered.

**Discussion:**

All assessments and the setting in total were tolerated well by participants and caregivers. Procedures were adapted to individual needs of the participants.

**Conclusion:**

All its components and the setting in total are absolutely feasible in the specific population of FXS individuals. Losses during consenting and recruiting have to be planned as well as high amounts of interindividual variances have to be taken into account.

## Introduction

The Fragile X Syndrome (FXS) is a so-called rare disease (OMIM 300624)—being the most common form of inherited mental retardation (1, 2) with a worldwide prevalence of 1:4000 boys and 1:6000 girls at school age (3). It is caused by a trinucleotide expansion of CGG polymorphism which leads to a hypermethylation of the FMR1 gene on the X chromosome (4). With more than 200 CGG repeats, the production of the Fragile X Mental Retardation Protein (FMRP) is absent (so-called full mutation). FMRP is involved in the regulation of protein synthesis and essential for typical brain development (5).

Sleep disturbances are common throughout different age groups of FXS patients (6–8). Parents report sleep problems in 32 up to 77% of children with FXS (6, 8), of which up to 64% are given some kind of sleep medication (7).

Despite the scope of the problem, we found only one study assessing sleep macroarchitecture objectively in FXS by using polysomnography. Miano et al. (9) examined a cohort of 14 FXS males who were observed for two nights in the sleep lab (9).

Different neuroendocrinologic parameters seem to be altered in FXS with respect to sleep and psychopathology. Interactions in symptomatology are controversially discussed (10–16). The role of melatonin in neuroprotection, cognitive, and learning disability has been discussed for FXS (17). In several studies, melatonin treatment in child and adolescent neuropsychiatry has been considered (17–20) and concrete recommendations for treatment were given (15–18, 20–22). For FXS however, we found only two studies directly assessing melatonin profiles (23, 24). Inhomogeneous results of both studies, however, can be explained by different samples (age), methods, and settings of assessment.

In sum, disturbances of sleep and circadian rhythms seem to common in FXS, but objective data is lacking. A major obstacle to obtain objective data might be the lessened compliance caused by cognitive and behavioral difficulties in FXS individuals (25). The aim of this study was to prove the feasibility of a complex multi-modal assessment of sleep and circadian rhythmicity considering the special needs of FXS individuals.

## Methods and Sample

**Figure 1:**
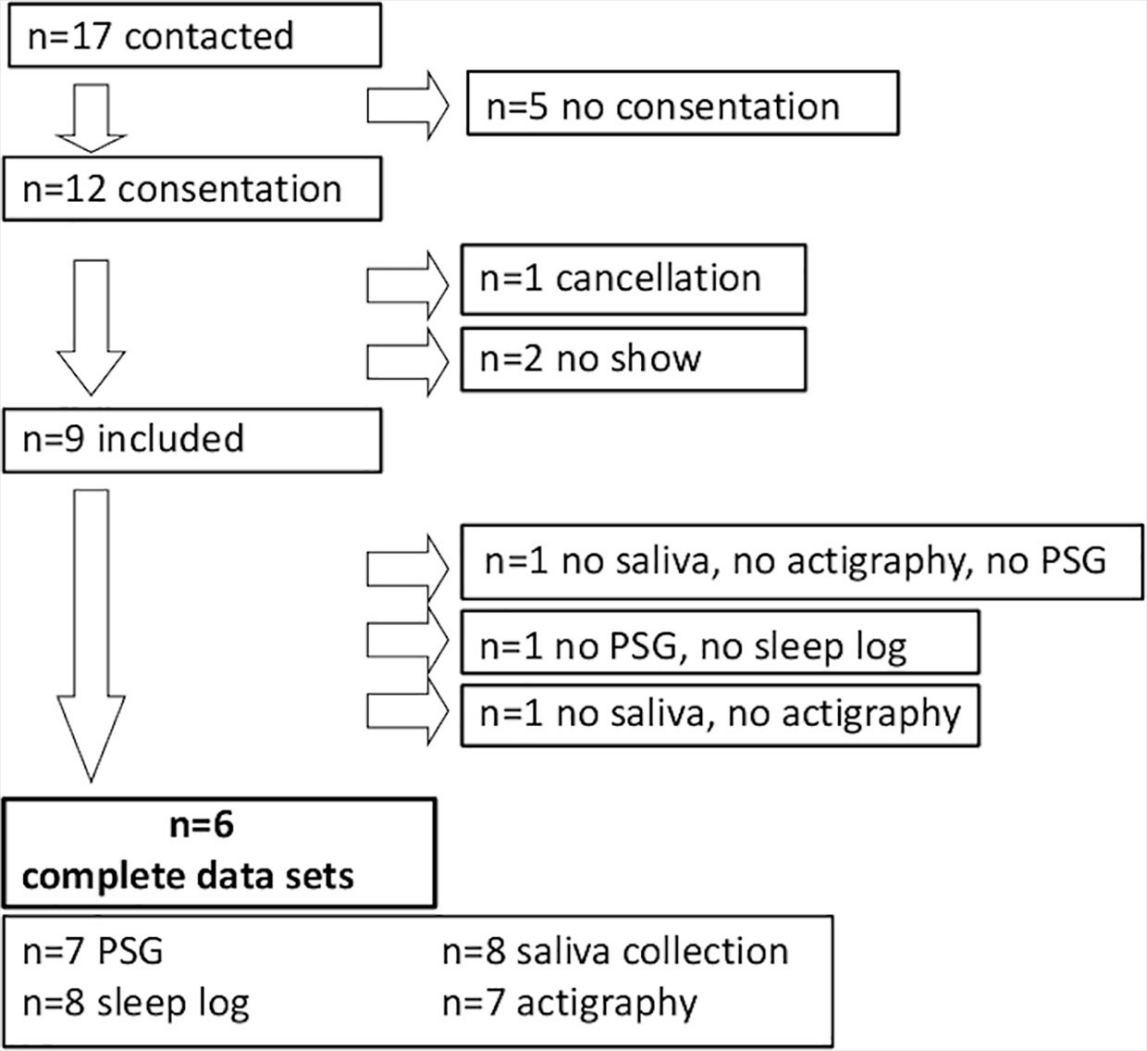
Flowchart.

The study design was approved by the ethics committee of Rostock University. Seventeen subjects with full mutation of the FMR1 gene were contacted by the treating physician and extensively informed about aims and setting of this study. After a meeting of subjects, caregivers (meaning parents and professional caregivers), and therapists, 12 subjects gave informed consent to participate after a time for consideration. Parents of one subject withdrew consent afterward and two participants did not show up for assessment. A sample of nine subjects was assessed (see flow chart in **Figure 1**) of which six complete data sets were gained. n = 7 PSGs, n = 8 sleep logs, n = 8 saliva profiles, n = 7 actigraphy data sets were collected. None of the participants got any kind of medical intervention or medication throughout the study.

### Sleep Environment

Sleep environment was documented. Investigations were realized at our hospital (n = 1) or at home in two centers in North and South Germany. Subjects and families chose by themselves whether assessments should take place at our hospital or at home. As the equipment was mobile it could be used at any location. If participants were assessed at home a researcher visited them there.

### Sleep Log

Sleep logs were kept for one week minimum and were filled out by caregivers. We asked for the time the subject went to bed, for bedtime routines, intake of medication, recuperation effects, sleep latency, WASO (wake after sleep onset) (number and time), time of sleep termination, and time of getting up. Global parameters of time in bed, total sleep time, variability of sleep duration, variability of sleep latency, variability of sleep onset, and variability of sleep termination were calculated.

### Polysomnography

Prior the investigation, the PSG hardware was presented to the subjects. That included a head box dummy that a contact person or the participant himself could wear for some days, this way adapting to it. All PSGs were realized as one-night measurements. For polysomnography, we used the following electrodes and sensors: EEG: F3, F4, C3, C4, CZ (as reference), O1, O2, A1, A2, and FpZ (as ground), EOG one site, EMG: chin, tibialis anterior muscle right (TAR) and left (TAL), ECG, thoracic and abdominal belt, flow, and position sensor. The system used was the Somnoscreen plus^©^ (SOMNOmedics GmbH, Randersacker, Germany). Data were collected and transferred *via* Bluetooth, with no electric wires between head box (located at participants chest), camera, or recording laptop being necessary. That gave participant the opportunity to move around without restrictions. Analyses were conducted by educated and experienced staff. Standard values were computed *via* the Domino^©^ software (SOMNOmedics GmbH, Randersacker, Germany).

### Saliva Samples

Sampling times were selected in relation to the current state of knowledge about endogenous melatonin secretion profile: 08:00 a.m., 12:00 a.m., 04:00 p.m., 06:00 p.m., 07:00 p.m., 08:00 p.m., 10:00 p.m., 00:00 p.m., 04:00 a.m., 08:00 a.m. Subjects were asked to refrain from eating or drinking at least 20 min before collection. Night samples were taken under dim light conditions while the subject stayed in bed. Light exposure prior and during sampling was measured by the light sensor of the actigraph (see **Figure 5**).

When sampling took place in the subject´s familiar environment, caregivers were extensively trained in the sampling method and were given a manual. If needed, they could contact the researcher 24/7. If it was regarded necessary participants were trained in spitting by a speech therapist before the assessment. Saliva samples were collected in a cup and then transferred to an Eppendorf tube. The samples were then stored in the domestic freezer until they were transferred to the laboratory cooled on dry ice. Melatonin was determined in saliva using a radioimmunoassay (Melatonin Saliva Direct RIA, DRG, Cat. RIA-5503) according to the manufacturer´s protocol. Samples (500 µl) were added with 25 µl of Enzyme. After 1 h of incubation, 50 µl Assay Buffer, 50 µl Adjustment Buffer, 25 µl ^125^I Melatonin and 25 µl Melatonin Antiserum were added into the tubes. The probes were incubated for 20–24 h at room temperature. Precipitation reagent (1000 µl) was added. After vortexing, the probes were incubated for 20 min at 2–8°C and then centrifuged for 20 min at 3000 × g at 2–8°C. The supernatant was decanted and the tubes were dried for 2 min. Radiation was counted for 1 min in the Berthold Technologies LB2111 Multi Crystal Gamma Counter, combined with the data Station LBIS 501 software from Berthold technologies. Each series of measurement was performed with its own calibration curve. The calibration curve was determined by using a smoothed cubic spline. The limit of detection was 1.4 pg/ml.

The Cortisol levels were measured using ECLIA-Technology (cobas e411, Roche, Penzberg, Germany) and Immunoassay for the *in vitro* quantitative determination of cortisol in human saliva (Roche, Penzberg Germany, Cat. 06687733 190). The limit of detection was 1.5 nmol/l. Calibrations and control measurement were performed according to regulations of DAkkS.

Both hormones melatonin and cortisol were examined in each sample, with priority on melatonin if the amount of saliva was too small to detect both.

### Actigraphy

The actigraph is a small, light-weight, wrist-worn monitor looking like a small watch. It includes an accelerometer (piezo element) and a light sensor to count motor activity and light exposure. Reading the data and charging the battery can be realized *via* USB. Participants were asked to wear the actigraph 24 h for at least 7 days (week and weekend) on the wrist of the non-dominant hand. The devices we used are waterproof, so they didn´t have to be removed while showering or bathing. Collected data was stored on a laptop and later transferred to a data base. Light exposure and movements were summarized and averaged for the period of 1 min. For our analysis, we enlarged the periods to 1 h. Interdaily stability and intradaily variability were computed directly from raw activity data according to the method used by Witting et al. (26). Both parameters are not dependent on *a priori* models of the data waveform (27). Interdaily stability scaled from 0 to 1 is a measure of consistency of circadian patterns (motor activity, light exposure) from one day to the next. It indicates the strength of coupling between a 24-h rhythm to an environmental zeitgeber. High intradaily variability is a measure for the hour-to-hour changes in activity and describes transitions between rest and activity. It also indicates nighttime activity or daytime resting.

Sleep scoring and awakening scoring was conducted with the Actiware^©^ v5.59 software (Phillips Respironics GmbH, Hamburg, Germany). An epoch was considered as “awake” when the sum of activity counts for the eligible epoch and the two succeeding and preceeding neighbor epochs were higher than 40. The formula for the activity count sum calculation was:

activity count = epoch_−2_ *1/25 + epoch_−1_*1/5 + epoch + epoch_+1_*1/5 + epoch_+2_*1/25. The sampling rate (epoch length) was 60 set for seconds. Movement data was collected over 7 successive days. As a movement detector the Motionwatch^©^ 8 (CamnTech, Cambridge, UK) was used. The sampling rate (epoch length) was 60 set for seconds. Movement data was collected over 7 successive days.

### Statistics

Cosinor analysis is a well-established statistical method to describe circadian rhythms under the *a priori* assumption about the cosinus-like waveform of the activity data. A cosinor curve with a 24-h period was fitted to the collected data using the least-squares method. Various parameters, indicating the circadian rhythm, such as period, amplitude, acrophase, mesor and percent rhythmicity can be derived from the cosinor function itself (28).

## Results

Complete data sets were assessed for one female and five males with a mean age 14.87 years (SD 4.12, range 8 to 20 years) and an averaged BMI of 25.90 (SD 4.44, range 18.08 to 31.64) (see **Table 1**). In no case, the investigation was interrupted or cancelled during the assessment.

**Table 1:** Cohort: descriptive statistics.

Age (years)			BMI (kg/m^2^)			Sex
Mean	SD	Range	Mean	SD	Range	1 f
14.87	4.12	8 to 20	25.9	4.44	18.08 to 31.64	5 m

SD, standard deviation; BMI, body mass index; kg, kilogram; m^2^, square meters; f, female; m, male.

### Sleep Environment

All six subjects slept in their own bed. Five had their own room, one shared the room with a sibling. For four subjects evening rituals or fix routines including bedding and pyjamas were observed. One participant did not show any sleep hygiene or evening routine and consumed media (TV and cell phone) in bed without parental regulation.

### Sleep Log

The sleep log was filled out for all of the subjects. The recuperation effect of sleep (1 - very much, 2 - somewhat, 3 - middle, 4 - hardly, 5 - not at all) was reported as “very much” by five and with “middle” by one participant. No medication was reported for all participants. Three participants reported about taking naps during day time, ranging from 10 min up to 2 h.

For mean values of total sleep time, variability of sleep duration, sleep latency, variability of sleep latency, variability of sleep onset and variability of sleep termination see **Table 2**.

**Table 2:** Sleep log data.

	FXS (n=6)	
	Mean	SD
Age	14.87	4.12
TST (h)	9.72	0.81
Variability of sleep duration (h)	0.77	0.71
Sleep latency (min)	8.69	6.57
Variability of sleep latency (min)	7.67	7.31
Number of night wake episodes	1.00	1.30
Total night awake period (min)	2.91	3.66
Variability of sleep onset (h)	0.35	0.19
Variability of sleep termination (h)	0.49	0.27

SD, standard deviation; FXS, fragile X syndrome; TST, total sleep time; h, hour; min, minute.

### Polysomnography

Wearing the hardware and the investigation itself was tolerated well by all participants. The installation of all sensors takes a lot of time and can be experienced as stressful by the participants. Therefore, the time budget was planned with options for breaks and deflection, running between 1 and 2 h. A tablet or TV was used to distract participants during the time of installation. The dummy of the head box was received positively. Three subjects lost or removed the flow sensor throughout the night. No epileptic activity was determined in our group. For mean values and standard deviation see **Table 3**.

**Table 3:** PSG data.

	FXS (n = 6) (1)	
	Mean	SD
Age	14.87	4.12
TIB (min)	518.80	59.22
TST (min)	399.08	85.92
Sleep efficiency	78.63	19.95
Sleep latency	32.30	27.47
REM latency	179.17	79.24
SPT (min)	465.20	44.70
Awakening/h	0.68	0.46
WASO (% SPT)	18.78	23.30
REM (%)	14.43	2.63
N1 (%)	14.38	10.17
N2 (%)	46.12	3.20
N3 (%)	25.03	8.38

SD, standard deviation; FXS, fragile X syndrome; TIB, time in bed; REM, rapid eye movement (sleep state); TST, total sleep time; SPT, sleep period time; WASO, wake after sleep onset; N1, N2, N3, sleep states.

### Saliva Samples

**Figure 2:**
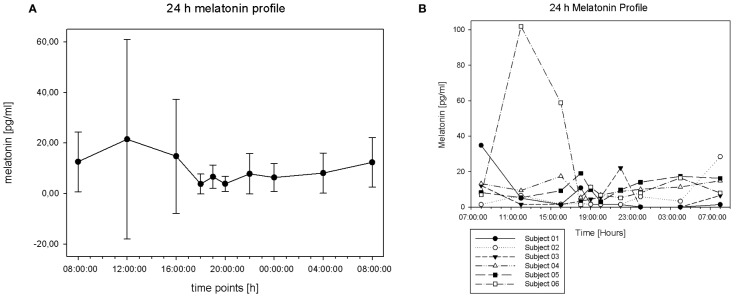
**(A)** 24-h melatonin profile (mean and SD). **(B)** Individual 24-h melatonin curves.

Sampling was tolerated well by all participants. During evening and night-time, the amount of saliva was somewhat reduced. In total, the data density was sufficient for melatonin, but less so for cortisol. Dim light melatonin onset (DLMO) can be defined as the period of time when melatonin concentration reaches 4 pg/ml or when concentration reaches doubled basal value. In our group, we found high melatonin values (above 4 pg/ml) but did not find an increase to doubled basal level in the group mean values (see **Figure 2A**). Surprisingly, we found the peak concentration around noon, with high values between the morning and afternoon, but not during the evening hours. As displayed in **Figures 2A, B** there levels vary a lot at all points of measurement, but especially in samples taken at 12:00 a.m. (mean 21.48 pg/ml, SD 39,49) and at 04:00 p.m. (mean 14.73 pg/ml, SD 22.57). Those results are mainly caused by the profile of subject number 6, as displayed in the individual 24 h melatonin trajectories (**Figure 2B**). Some participants show individual peaks of concentration reaching the doubled basal level. Subject 1 peaks around 6:00 p.m., subject 3 around 10:00 p.m., subject 4 around 7:00 p.m., and subject 5 around 4:00 p.m. Subject 6 shows high values around noon with a following decrease in the afternoon and a second, but much smoother increase in the evening. Some individuals show peak concentration in the afternoon/evening time (subjects 1, 3, 4, 5, 6). Others have their peak in the morning (subjects 1, 2, 3) or show two peaks (subjects 1, 3, 6).

**Figure 3:**
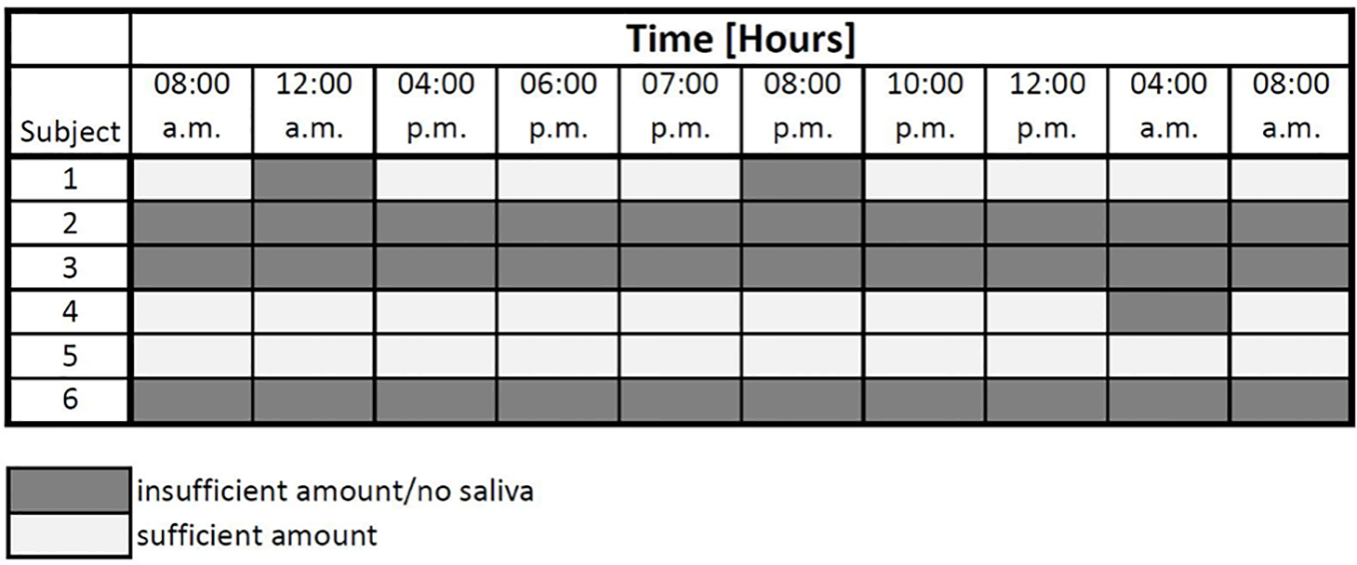
Sufficiency of saliva for cortisol measurement.

**Figure 3** displays the amount of saliva for cortisol measurement. Unfortunately, the amount of data was not sufficient in our study to be analyzed *via* the cosinor fit method. This is mostly due to a lack of material in the samples taken. For three individuals samples were too small to be analyzed effectively.

In sum, patterns of hormone secretion during the day vary widely across individuals and time. A major problem occurred with the sampling of sufficient amounts of saliva for two targets.

### Actigraphy

Wearing the actigraph was tolerated well by all of the subjects. Only two of the nine subjects included in the study did not want to wear an actigraph over the period of time. In both cases, there were concerns against the long duration of the investigation rather than the method itself.

**Figure 4:**
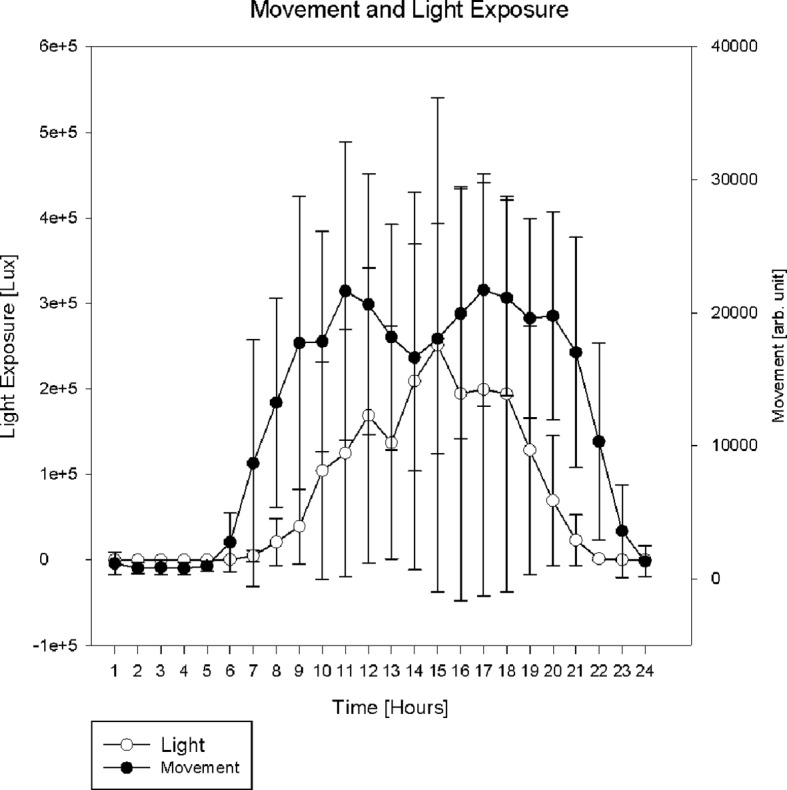
. Actigraphy data: group mean and standard deviation of light exposure and movement.

To improve compliance in participants, the researcher or a close contact person also wore an actigraph (dummy) during investigation when considered appropriate. For group means and standard deviations of light exposure and movement per hour, see **Figure 4**. For calculated time in bed, total sleep time, sleep latency, sleep efficiency, and WASO; see **Table 4**.

**Table 4:** Approximate sleep macro architecture based on actigraphy data.

	Mean	SD
TIB (min)	536.00	23.32
TST (min)	446.13	17.56
Sleep latency (min)	12.67	6.22
Sleep efficiency (%)	83.30	2.07
WASO (min)	64.75	8.92

SD, standard deviation; TIB, time in bed; REM, rapid eye movement (sleep state); TST, total sleep time; WASO, wake after sleep onset.

The averages of interdaily stabilities for all participants were 0.35 (SD 0.28) for motor activity and 0.24 (SD 0.19) for light exposure. Intradaily variabilities were 0.61 (SD 0.14) for motor activity and 0.94 (SD 0.48) for light exposure.

**Figure 5:**
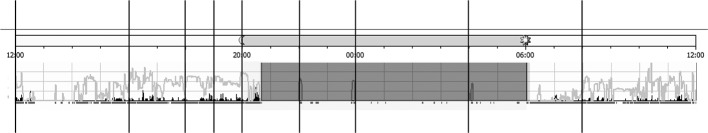
Actigraphy data: control of saliva sampling for light exposure and motor activity.

**Figure 6:**
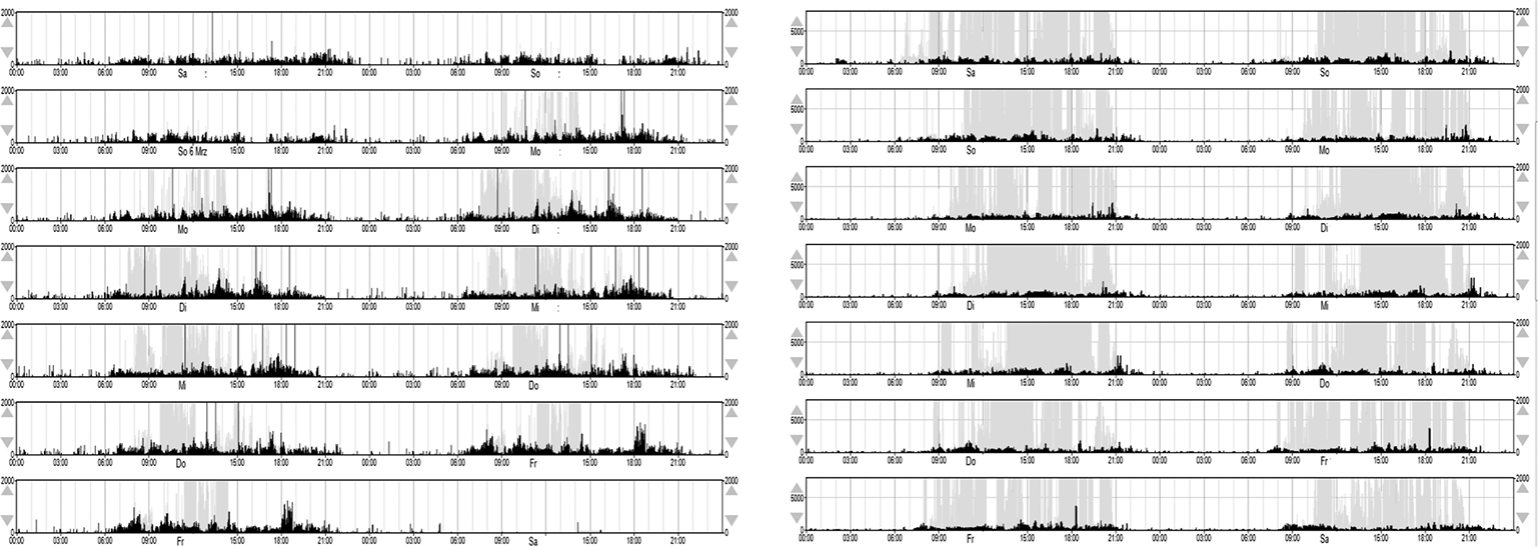
Actigraphy data: double plots of two individuals.

**Figure 5** displays motor activity and light exposure for a single participant for the day of saliva sampling. That way both parameters were controlled at times of sampling (vertical lines). **Figure 6** shows double plots of movement and light of two different FXS individuals over a time period of one week. In the plot on the left side variances of light exposure (light grey) and motor activity (black) are displayed. With this method the stability of daily routines (subjectively described by the sleep log) can be depicted objectively. All single cosinor fits reached the level of significance for light exposure and motor activity data (p 0,000) for the period of 24 h, averaged over for 7 days. For mean amplitude, mean mesor, percent rhythmicity of light exposure, and motor activity; see **Table 5**. Mean acrophase was around 02:00 p.m. for light exposure and 03:00 p.m. for motor activity.

**Table 5:** Cosinor analysis of actigraphy data.

	Motor activity		Light exposure	
	Mean	SD	Mean	SD
Amplitude	11040.00	4837.71	116180.83	121024.06
Acrophase	14.48	1.48	13.82	1.65
Mesor	12273.33	5065.75	78037.00	80847.91
Percent Rhythm	74.62	15.63	65.69	16.40

SD, standard deviation.

## Discussion

A ratio of 12 participants consenting to nine participants included signals an attrition of about 33%. Thus, future studies should plan an oversampling of informed consents of about one third. Another third was lost during assessments (nine included/six complete data sets) with resisting to wear actiwatches for a whole week being the major reason for dropping out. However, no investigation was interrupted or cancelled during the assessment when certain individual adaptations were undertaken. In general, we regard it feasible to collect complex data on sleep and circadian rhythms in individuals with FXS. Feasibility should be explained in greater detail for different variables.

### Sleep Environment

Nearly all subjects lived and slept in sleep supportive surroundings. There were families who strictly followed the same routines every day, including light exposure, eating, etc. For one girl, however, no sleep hygiene and routines were established.

### Sleep Log

Naps were common for about one third of our participants (2 out of 6). In further studies, daily sleep routines need to be addressed along with the night sleep. The effect of sleep recuperation was assessed as very good to middle. Sleep log data represent the subjective opinion of caregivers about individual sleep (quality) which can be objectified by actigraphy. Differences in both parameters can indicate caregivers burden.

### Polysomnography

In our study, seven out of nine participants underwent the PSG and tolerated it well. We successfully reduced rejection and burden, and enlarged compliance by 1) a dummy of the head box worn by the participant himself or by a close contact person for some time, 2) allowing the participant to use the tablet or watch TV during installation of the PSG electrodes, and 3) to move around freely. Compared to PSGs done at the lab, in-home investigations gave us the opportunity to observe daily routines and direct sleep environment of each individual. Thus, we decided to offer both settings to choose from. Miano et al. (9) conducted PSG under lab conditions and added one adaption night to reduce the first night effect (9). In order to minimize participants' burden, we decided to realize just one overnight measurement in our study. For children and adolescents, single-night PSG gave us also the opportunity to compare our data to normative values published by Scholle et al. (29) for a similar setting (29).

Despite seizures are common in FXS (30), we did not find any signs of it in our sample. The literature suggests rates of epilepsy in 10–20% and EEG anomalies in up to 74% of FXS-subjects (31). In order not to overlook signals indicating seizures, we decided to use a 10 channel EEG montage instead of the AASM recommended 3 channel derivation (32), which was well tolerated.

According to a study by (33), FXS individuals are at increased risk for obstructive sleep apnea syndrome (OSAS). In order to detect sleep disordered breathing, we decided to use a nasal probe with a pressure sensor (flow) and thoracic and abdominal belts. Belts were much better tolerated than the flow sensor.

### Saliva Samples

We decided to let subjects spit into a sterile cup. This method was well tolerated and associated with low nighttime disturbance of the participants (see **Figure 5**). Both samples we compared our data with used radioimmunoassay (23, 24), as it was used in this study. Our data reveal a high amount of variance in levels of melatonin concentration at different time points (see **Figure 2**). Gould et al. did measure participants during a two days period doubling the amount of data per time point, but still found high amounts of variance within their data. For the melatonin peak with mean concentration of 169.87 pg/ml the SD was reported with 108.94 (24), which appears to be very similar to our findings.

In our cohort, we detected the melatonin peak concentration around noon (see **Figure 2A**). This contrasts the expected (physiological) profile with an increase of concentration during evening hours (DLMO) and high values during nighttime (34). High midday values in our study are associated with a high interindividual variance especially in the 12:00 a.m. and the 04:00 p.m. samples. The midday peak concentration is mainly caused by the oldest subject (20 years) in our group (subject 6, see **Figure 2B**) and is associated with extensive naps (2 h) every day (see sleep log section) in the afternoon. We found similarly high melatonin values during daytime in a 49-year-old woman, who did not take part in the complete setting. The high melatonin values around midday for two grown-up persons we suggest to be a hypothetic characteristic of adults with FXS. However, they might as well represent individual deviations. This question needs to be answered by further research. In contrast, O´Hare et al. (23) did not find any significant increase of serum melatonin levels during a 24-h period (sampling every 3 h) in an adult (mean age 59, range 47 to 69) FXS sample. The investigation took place in the hospital and melatonin examination was realized with blood drain *via* an intravenous cannula inserted at midday (23). Arguably, the lack of increase in O´Hare´s publication might be a result of the lab conditions. Gould et al. (24) investigated melatonin *via* in-home saliva sampling and described an increase of the melatonin level at 08:00 p.m. with a decrease at 08:00 a.m., a maximum peak at 02:00 a.m., and generally higher levels in their group of n = 13 FXS boys (mean age 8.04; SD 1.65; range 4.7 to 11.0 years) compared to the control group (24). The mean age in our group was very similar (14.87 years with just one 8 year old subject). Unfortunately, the amount of saliva from this young boy was not sufficient in the relevant samples (10:00 p.m., 00:00 p.m., 04:00 a.m. and second 08:00 a.m.) as it would be needed to compare the studies' data to our participant. However, the first 08:00 a.m. level (34.8 pg/ml) for the 8-year-old is much higher than the following ones (12:00 a.m.: 5.0 pg/ml) and all the levels during daytime. This refers to high levels during nighttime similar to Gould et al. (24).

The decrease of melatonin levels from childhood to adulthood in FXS might not only be a general progressive decrease with age (24). High concentrations in infants and prepubertal children (35), may suggest a drift in circadian profile of secretion. The finding of high melatonin values around midday in FXS adults under home conditions might be an important result and may have some potential for therapy. Treatments may include the organization of daytime structure and sleep interventions up to melatonin administration. To offer a nap might reduce “abnormal” and challenging behavior in some patients and increase quality of life—for the subject itself but also for the caring system. However, persistent high melatonin levels could be caused by food (36) or slow melatonin metabolism, itself perhaps caused by CYP1A2 gene polymorphism which might be associated with autism (37, 38). Both factors, food and CYP1A2 polymorphism and their interaction, should be addressed in further investigations.

FXS is often accompanied by psychiatric symptoms, such as ADHD-like behavior, ASD and challenging behavior (39) which often leads to pharmacological treatment (40). Gender differences in FXS´ psychopathology are well established. Females with FXS show more mood disorders than healthy controls. Males with FXS show more ADHD-like and challenging behavior (41–43). ADHD itself is strongly associated with a disturbed circadian rhythmicity (44–46). The role of the HPA-Axis in FXS in general as well as in the psychopathology of the syndrome (ADHD, ASD, depressive symptoms, challenging behavior etc.) has often been described (10–14). However, the association of the cortisol level and the manifestation of autistic behavior is discussed controversially in this context (11, 13). Therefore, a sufficient examination of cortisol in saliva samples is recommended. Additional samples for the morning and early daytime should be taken to clarify these mechanisms. An extensive neuropsychiatric examination, including (mental) health, development, and caregivers burden should be useful. Cosinor analyses on hormone data should be useful to display the interplay of neuroendocrine and psychiatric aspects in FXS.

### Actigraphy

The combination of saliva sampling with actimetry gives the researcher the opportunity to control time points and light conditions (see **Figure 5**). Constant light conditions during daytime, dim light and minimal movement during nighttime sampling become visible and controllable. As visible, the taking of samples did not disturb the night sleep. Constant routines, as indicated by sleep logs can be objectified by double plots for weeks, as shown in **Figure 6**.

In our sample, rhythmicity in the group is not very stable (IS < 0.5) and quite fragmented (IV > 0.9) for a period of one week. Instability of circadian rhythmicity and variability of sleep patterns, however, can be risk factors in family and caring systems.

In our small cohort single cosinor analyses were computed for each participant separately. For further analysis and correlation with other data, a groupwise cosinor analysis would be useful.

## Conclusion

Polysomnography in our setting was well tolerated by participants and caregivers. It requires high efforts in time, logistic, and personal engagement. With a mobile system, overnight PSG investigations can be realized at the hospital or at home. Compliance can be enhanced by several measures tailored to the FXS population. In order to detect abnormal EEG-activity more than three electrodes should be used, which proved to be feasible. As the nasal probe was not tolerated by many of the participants, calculation of X-flow could be a useful and less invasive alternative to determine OSAS in FXS. PSG data will give us the opportunity to correlate objective sleep data with data on (mental) health and the subjects' and caregiver's burden. High variances, especially for hormone data, require large samples.

In-home, saliva collection proved to be a good method for this special group of participants. The best way to collect saliva from children with intellectual disability was to let the subjects spit into a sterile cup and then transfer saliva to an Eppendorf tube. Measuring hormones, melatonin, and cortisol from one saliva sample has led to comparatively poor data. However, the measurement of a full melatonin profile is essential for therapeutic considerations. For cortisol, additional saliva samples in the morning could be useful. To assess whether the melatonin peak at noon is a phenomenon typical for FXS in total the impact of age needs to be checked in a larger cohort of different ages. High melatonin concentration around midday in FXS (adults) might have important implications for treatment and daily life. Further investigations of melatonin profiles should include a sufficient amount of subjects in different age groups and address both food intake and CYP1A2 gene polymorphism. Actigraphy is a minimally invasive and well tolerated opportunity to measure circadian rhythmicity over a longer period of time. To find typical FXS patterns in sleep and circadian rhythmicity, an investigation within a big cohort and a comparison to a matched group (e.g., in sleep routines) would be necessary.

Therefore, the first author is in contact with the German Fragile X family support association Interessengemeinschaft Fragiles-X e.V. Germanys' biggest organization of affected people and their families, counting about 1000 FXS affected members, agreed to help to realize a bigger project. Extrapolating our results in recruitment, 500 to 550 individuals could be integrated and 300 to 350 complete data sets could be collected. An investigation with this number of participants all over Germany would be a logistic mammoth task, but would offer the opportunity to understand the structure of sleep problems. The optimal way to fully understand the role of sleep and circadian rhythms in the development of FXS of course, would be a longitudinal study over a time period of years.

In sum, disturbances of sleep and circadian rhythms are common in FXS. So are intellectual disability and psychiatric symptoms. Häßler et al. (47) point out the substantial burden of psychiatric and mental problems in patients with FXS of all age groups and recommend the initiation of an early psychiatric expert diagnosis with subsequent multimodal and multi-professional individualized management (47). There are indications for sleep and circadian rhythmicity playing an important role in this context. This study has proven the feasibility of a complex setting for the assessment of sleep and circadian rhythmicity considering the special needs of FXS individuals.

## Data Availability Statement

All datasets generated for this study are included in the article/supplementary material.

## Ethics Statement

The studies involving human participants were reviewed and approved by Ethics Committee of Rostock University Medical Center. Written informed consent to participate in this study was provided by the participants' legal guardian/next of kin.

## Author Contributions

All authors listed were part of data collection and/or publication.

## Conflict of Interest

FH was employed by the company GGP-Gruppe (Gesellschaft für Gesundheit und Pädagogik mbH), a corporation for health and education.

The remaining authors declare that the research was conducted in the absence of any commercial or financial relationships that could be construed as a potential conflict of interest.
